# The pay-off of hypsodonty - timing and dynamics of crown growth and wear in molars of Soay sheep

**DOI:** 10.1186/s12862-018-1332-9

**Published:** 2018-12-29

**Authors:** Carsten Witzel, Uwe Kierdorf, Kai Frölich, Horst Kierdorf

**Affiliations:** 1 Department of Biology, University of Hildesheim, Universitätsplatz 1, 31141 Hildesheim, Germany; 2 Tierpark Arche Warder e.V, Langwedeler Weg 11, 24646 Warder, Germany

**Keywords:** Hypsodonty, Enamel, Tooth wear, Fluorochrome labeling, Tooth development, Longevity, Adaptation

## Abstract

**Background:**

Several lineages of herbivorous mammals have evolved hypsodont cheek teeth to increase the functional lifespan of their dentition. While the selective drivers of this trend and the developmental processes involved have been studied in greater detail, thus far no quantitative information is available on the relationship between additional investment into tooth growth and the resulting extension of the functional period of these teeth. To achieve this, we performed a detailed analysis of molar crown growth in known-age Soay sheep repeatedly injected with different fluorochromes.

**Results:**

Our study revealed that in sheep molars especially the formation of the crown base portion is prolonged in comparison with other herbivorous artiodactyl species. Our results demonstrate that growth of the crown base accounted for more than half of the total crown formation time (CFT) of the anterior lobes of the first (approx. 220 days of total CFT of 300 days), second (approx. 260 of 460 days) and third (approx. 300 of at least 520 days) molars, and that the formation of this crown portion occurred largely after the teeth had already reached functional occlusion. By combining data on wear-related changes in crown morphology from the literature with the reconstructed additional investment into the crown base portion, it was possible to relate this additional investment to a prolongation of the functional periods of the molars ranging from 4 years in the M_1_ to 6 years in the M_3_.

**Conclusions:**

Our results allow to establish a quantitative link between an additional investment into molar crown growth of sheep and the extension of the functional period of these teeth. The reported findings enable an assessment of the adaptive value, in terms of increased longevity, of an additional investment into crown elongation in a mammalian herbivore.

## Background

As mammals typically possess only two generations of teeth (diphyodont condition), there is a considerable selective pressure on maintaining the functional durability of their teeth [1]. The wear to which teeth are exposed can be subdivided into attrition, i.e. tooth to tooth wear, and abrasion, i.e. wear by mechanical factors other than tooth to tooth contact [2]. The dentition of herbivorous mammals, grazers in particular, is exposed to heavy wear, whose intensity varies with the silica content of the consumed plant material and its contamination with grit [3–7]. During their evolution, herbivorous mammals followed different adaptive avenues to increase the functional lifespan of their dentitions [4]. One such adaptation is the increase in relative tooth height, i.e. the formation of high-crowned teeth. The evolution of such a hypsodont condition from an ancestral brachydont state occurred in several mammalian lineages [4, 8, 9]. The degree of hypsodonty achieved by a particular species can be viewed as reflecting the amount of dental wear to which it has adapted during evolution [9, 10]. Developmentally, hypsodonty is the result of a prolonged period of tooth crown formation mediated by specific signaling molecules, implying a prolonged presence of a stem cell niche at the apical rim of the enamel organ along with the capability of the derived cells to differentiate into secretory ameloblasts [11–14].

Analyses of herbivorous species from fossil mammal assemblages across Europe and Central Asia indicate an increase in hypsodonty with the occurrence of increasingly arid climatic conditions during the Miocene that resulted in the spreading of open, grass-dominated landscapes [3, 15, 16]. The development of higher crowns in cheek teeth thus constitutes a key innovation among grazers that co-evolved with the grassland vegetation [1, 17–19]. It is debated whether the grass dominated diet itself or its contamination with grit was the major driving force of this process [20]. The adaptive value of an increased crown height is demonstrated by the observation that among ruminants, hypsodont clades had higher speciation and diversification rates than non-hypsodont clades [21].

Hypsodonty has been defined as a condition where teeth are higher than wide or long [22, 23]. Several types of hypsodonty have been distinguished on the basis of which portion of a (molar) tooth is elongated. White [23] used a straightforward classification by distinguishing between cusp hypsodonty, tooth base hypsodonty, and root hypsodonty. The latter condition involves developmental processes that differ from those that cause an increase in crown height [24]. The distinction between cuspal and basal crown portions is based on their position relative to the floor of enamel-bordered infoldings in the occlusal surface referred to as infundibula [25]. Crown areas located occlusal to the infundibular floor constitute cuspal, those located cervical to this landmark basal crown portions. An elongation of the tooth crown due to prolonged growth below the level of the infundibular floor is therefore designated as tooth base hypsodonty [23]. When the cuspal crown portions along with the infundibula are worn away in teeth exhibiting tooth base hypsodonty, enamel is located only at the outer circumference of the tooth crown. White [23] classified the molars of bovids, including the genus *Ovis*, as exhibiting cusp hypsodonty. However, other authors had already noted that sheep molars possess both, elongated cusp portions and elongated tooth bases [26, 27], thus combining cusp and tooth base hypsodonty.

Von Koenigswald [28] later proposed a classification system applicable to all tooth types. This system is based on identifying heterochronic shifts in tooth development, i.e. the prolongation of certain ontogenetic phases (formation of cusps, sidewall, cervix, or root) of tooth growth relative to others. According to this classification [28], the molars in the genus *Ovis* exhibit sidewall hypsodonty. Neither the classification system of White [23] nor that of von Koenigswald [28] quantifies the relative contribution of the different crown growth phases to the achieved degree of hypsodonty.

An extreme form of hypsodonty results from a complete suppression of root formation, as it occurs in ever-growing (hypselodont) teeth [8]. True hypselodont molars have evolved only very rarely in ungulates (e.g., *Elasmotherium* and some species of Notoungulata [4, 29, 30]), but it has been noted that root formation starts very late during odontogenesis in extant horses and some fossil horse species (e.g. *Pseudhipparion simpsoni*), and among ruminants in sheep (*Ovis aries*) and pronghorns (*Antilocapra americana*) [4, 26, 27, 31–34]. The latter condition has been termed incipient hypselodonty by Webb and Hulbert [26]. Late onset of root formation during odontogenesis has, however, also been described as a general feature of crown hypsodonty [35–37]. A diagnostic criterion for this condition is that the crown base of a tooth in functional occlusion lies below the alveolar crest of the jaw bone for a considerable period of time [35]. In horses, this crown portion is often referred to as the “reserve crown” [32, 38]. For sheep, no detailed data on the contribution of the elongated crown base to total crown height and the percentage of the total crown formation time needed for its formation are available so far.

A means to compare the degree of hypsodonty among different taxa is the calculation of a height to width ratio (HWR) mostly referred to as hypsodonty index (HI). Several ways of calculating HIs have been proposed, such as dividing height by width in third molars [37] or height by length in second molars [3]. Problems in determining HIs arise when the teeth become functional prior to the completion of crown formation. In such cases, a fully formed, but still unworn crown never exists, a condition present for example in sheep molars [26, 27, 39].

The aim of the present study is to provide a detailed analysis of molar crown growth in sheep. For this we studied mandibular molars of vitally labeled, known-age Soay sheep macroscopically and by light microscopy. We quantify, for the first time, the proportions of the total CFT needed for the formation of different crown portions and the related variation of crown growth rates. We further establish HWRs for mandibular molars in different growth and wear stages and discuss problems of comparing the data for sheep with those obtained in species with different crown growth patterns. By combining data from this study with those from the literature, we assess the relation between investment into crown growth of sheep molars and the pay-off in terms of a prolonged functional durability of the teeth and, in consequence, lifespan of the individual.

## Materials and methods

We analyzed the formation of different crown portions and wear-related changes in crown morphology of mandibular molars of Soay sheep. This is an unimproved breed that appears to be intermediate between modern domestic sheep and wild sheep in many aspects of its anatomy and physiology [40, 41].

Thirteen individuals (5 males, 8 females) from the Soay sheep herd at the Tierpark Arche Warder e.V. (Warder, Germany) were included in the study. All experimental procedures were performed in accordance with the current animal care regulations in Germany and with permission of the responsible veterinary authorities of the federal state of Schleswig-Holstein (Ministerium für Landwirtschaft, Umwelt und ländliche Räume des Landes Schleswig Holstein; Az. V312–72241.123-34). The experimental animals were earmarked and kept on pasture in an enclosure providing shelter huts from April to late October. During the remaining part of the year, they were kept in a stable and given hay and pelleted feed.

Eleven sheep were repeatedly injected with different fluorochromes. These animals were born between March 2009 and May 2012, and slaughtered (mechanical stunning using a bolt pistol followed by exsanguination) at different postnatal ages (69–785 days). The precise age at death (days after birth) is known for eight of the labeled individuals. For the other three, age at death is known within a range of ±10 days (Table 1). Nine sheep received injections of calcein (C; Sigma Aldrich, product no. C0875, buffered to pH 7) at a dosage of 8 mg/kg body weight, and of oxytetracycline (T; Serumwerk Bernburg AG, product no. 09932159) at a dosage of 80 mg/kg body weight in one to four labeling periods (for the injection schedule see Table 1). Two sheep were injected with three different fluorochromes, using calcein blue (CB; Sigma Aldrich, product no. M1255, buffered to pH 7) as the third fluorochrome at a dosage of 15 mg/kg body weight (Table 1). Finally, two unlabeled sheep that had died of unrelated causes during the study period were included in the analysis (Table 1).

**Table 1:** Sex and age-at-death composition of the studied Soay sheep, and postnatal ages in days at the dates of the injection of the different fluorochromes (C: calcein, T: tetracycline, CB: calcein blue). 0 = day of birth

Individual #, sex	Age at death (days)	Age (days) at fluorochrome injections (C: calcein, T: tetracycline, CB: calcein blue)
*79618*, male	69	0 C, 14 T, 28 C, 42 T, 56 C
*79756*, female	90	0 C, 14 T, 28 C, 42 T, 56 C
*79615*, male	327	0 C, 14 T, 28 C, 42 T, 56 C, 147 C, 161 T, 175 C, 196 T, 210 C
*79617*, female	327	0 C, 14 T, 28 C, 42 T, 56 C, 147 C, 161 T, 175 C, 196 T, 210 C
*22213*, male	410 (± 10)	96 T, 112 C, 133 CB, 154 C, 175 CB, 196 T, 217 CB, 238 T, 293 CB, 314 C, 333 C, 355 CB, 384 T
*22217*, male	410 (± 10)	96 T, 112 C, 133 CB, 154 C, 175 CB, 196 T, 217 CB, 238 T, 293 CB, 314 C, 333 C, 355 CB, 384 T
*79768*, female	472	6 C, 20 T, 34 C, 48 T, 62 C, 188 C, 202 T, 216 C, 237 T, 251 C, 377 C, 391 T, 405 C, 419 C, 433 T
*79675*, male	479	0 C, 14 T, 28 C, 42 T, 56 C, 195 C, 209 T, 223 C, 244 T, 258 C, 384 C, 398 T, 412 C, 426 C, 440 T
*79767*, female	671	0 C, 14 T, 28 C, 42 T, 56 C, 188 C, 202 T, 216 C, 237 T, 251 C, 377 C, 391 T, 405 C, 419 C, 433 T, 562 C, 576 T, 604 T
*79734*, female	700 (± 10)	0 C, 14 T, 28 C, 42 T, 56 C, 180 C, 194 T, 204 C, 229 T, 243 C, 369 C, 383 T, 397 C, 411 C, 425 T, 554 C, 568 T, 582 C
*79674*, female	785	0 C, 14 T, 28 C, 42 T, 56 C, 195 C, 209 T, 223 C, 244 T, 258 C, 384 C, 398 T, 412 C, 426 C, 440 T, 569 C, 583 T, 597 C, 611 T
*79634*, female	approx. 900	not labeled
*3357_28,* female	> 1800	not labeled

The heads of the animals were skinned, defleshed, macerated and defatted as described previously [42]. The cleaned and dried crania and mandibles were photographed and X-rayed prior to the removal of the left mandibular molars in the labeled animals (11 M_1_s, 9 M_2_s, and 7 M_3_s). Following photographic documentation, the teeth were embedded in epoxy resin (Biodur E12/E1, Biodur products, Heidelberg, Germany). The embedded teeth were sectioned axiobuccolingually through the anterior lobes as close as possible to the highest points of the protoconid and the metaconid. Ground sections for light microscopy (bright-field and fluorescence imaging) were prepared from the blocks as described previously [42, 43] and viewed in a Biozero 8000 inverted digital fluorescence microscope (Keyence, Osaka, Japan).

Scaled micrographs obtained using bright-field illumination and the different fluorescence lightings (filter specifications: calcein blue (CB) – excitation filter (ex) 365/50 nm band pass; dichroic mirror (dm) 395 nm; emission filter (em) 420 nm long pass; calcein (C) – ex 470/40 nm band pass; dm 495 nm; em 535/50 nm band pass; tetracycline (T) – ex 390/40 nm band pass; dm 452 nm; em 562/40 nm band pass; cf. [42]) were stitched with a stitching tool [44] for the Fiji freeware package (http://fiji.sc) and prepared as overlays. From these overlays or scaled macroscopic images in the case of unlabeled individuals, measurements of crown height (vertical distance between the highest point of the lingual anterior cusp (metaconid) to the apicalmost point of the buccal enamel) and crown width (greatest horizontal distance between the lingual and buccal crown flanks) were taken using the measuring tool of Fiji. These values were used for the calculation of the height to width ratio (HWR) whose maximum value in the M_3_ corresponds to the hypsodonty index (HI) according to Janis [37]. Molar wear rates (μm/day) were calculated from linear regressions of tooth height on age in days for teeth with fully formed crowns.

The fluorescent bands mark successive positions of the mineralization fronts in the dental hard tissues at the time of injection. These data and the state of dental development at the time of death were used for scoring different stages of tooth development. The following developmental stages were distinguished for the anterior lobes of the three mandibular molars: (1) start of mineralization, (2) completion (fusion) of the infundibular floor, and (3) completion of enamel formation bucally. For each postnatal age represented in the sample, it was scored whether the respective developmental stages had already been attained by the molars.

For calculation of daily enamel extension rates (EERs), the distance between consecutive labels was measured along the enamel dentin junction (EDJ) on the scaled micrographs. These values were then divided by the number of days between the respective fluorochrome injections. In order to attribute these daily rates to certain locations in the molar crowns, the maximum mean length of the EDJ was reconstructed at the buccal side for unworn and fully formed anterior lobes (a stage never really present) of the molars. For this, the distance from the tip of the dentin horn to the fluorescent band representing the calcein injection at day 56 was measured in unworn cusps of M_1_s. In M_1_s exhibiting wear, the distance between this fluorescent band and the crown-root-border (CRB) was measured. Summing up the mean values for both stretches yielded the reconstructed maximum mean length of the EDJ of the buccal flank of M_1_s. For M_2_s and M_3_s this procedure was also performed, using fluorescent bands that were present both in cusps exhibiting no or only minor wear and in already worn specimens. However, even in the M_3_ of the oldest labeled individual, crown formation had not yet been completed. This notwithstanding, the measured length of the EDJ in this specimen was used for reconstructing crown height in M_3_s. For each molar class, the reconstructed complete length of the EDJ was divided into quarters (1: cuspal/upper lateral, 2: mid lateral, 3: lower lateral, 4: cervical crown portion). Results for daily EER were grouped according to the location of the end-point for measurements, i.e. the apicalmost fluorescent band meeting the EDJ in the respective quarter. If more than one label was present in a quarter, a mean value was calculated for the EER.

The height of the crown portion located between the floor of the infundibulum and the apical enamel border of M_1_s and M_2_s was measured on the buccal side of the anterior lobe and is referred to as crown base height. In the case of the M_3_, the full height of the crown base portion could not be recorded, since crown formation was still ongoing in this region in the oldest animal injected with fluorochromes.

## Results

**Fig. 1:**
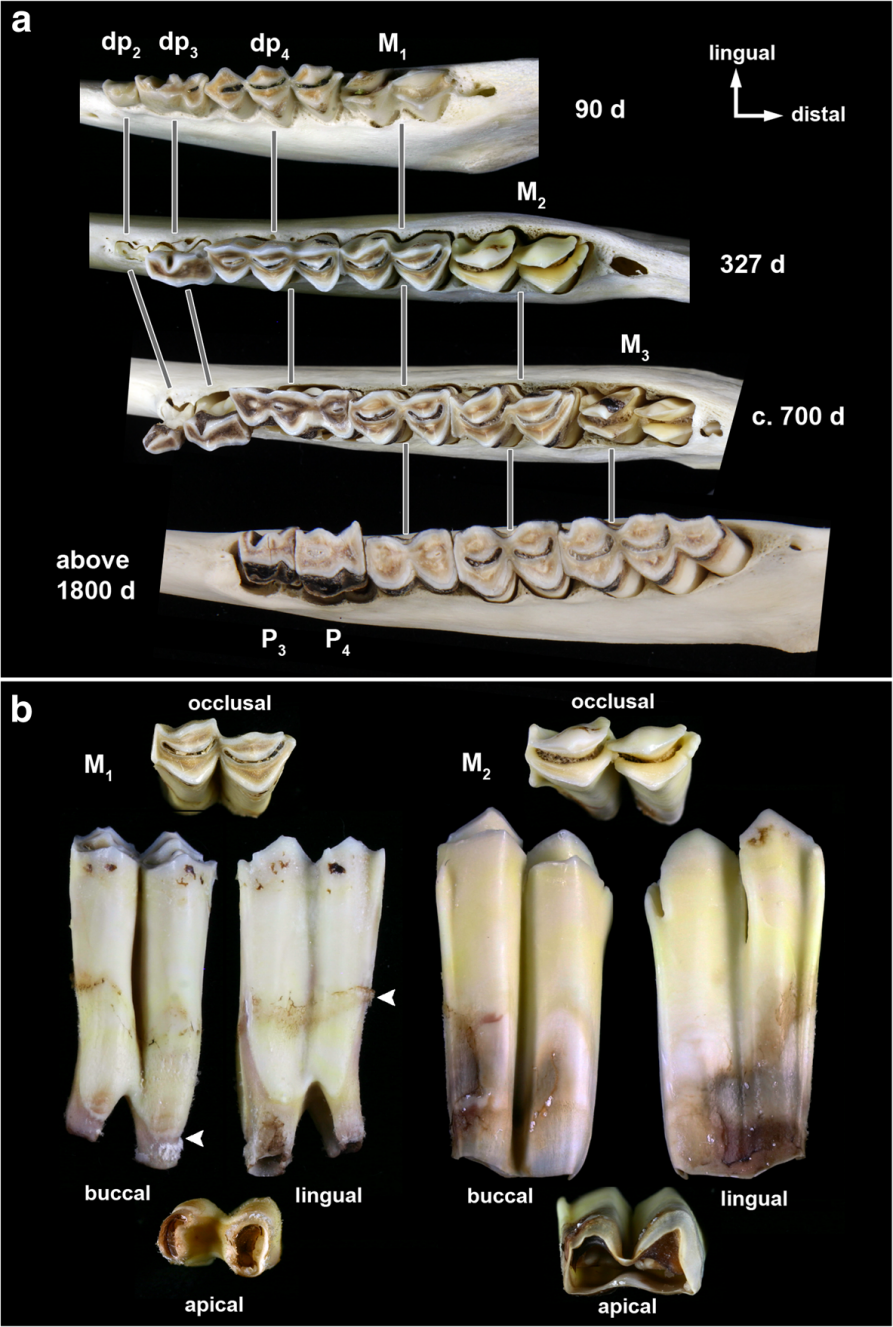
Mandibular cheek teeth of Soay sheep. **a**) Macrographs of left mandibular cheek tooth rows (occlusal view) of Soay sheep of different ages at death. Note that the P_2_ is congenitally absent in the oldest individual and the infundibulum has already been completely (anterior lobe) or largely (posterior lobe) worn away in the first molar. **b**) Macrographs of different views of the left mandibular M_1_ and M_2_ of individual # 79617, age at death 327 days. Arrowheads mark the cuspalmost and apicalmost positions of the crown root border in the M_1_

The mandibular molars of sheep erupt sequentially and typically come into wear prior to the eruption of the permanent premolars (Fig. 1a). However, eruption of the permanent premolars can sometimes overlap with that of the M_3_. Sheep molars exhibit the selenodont morphology characteristic for ruminants, with the buccal and lingual cusps separated by deep, funnel-shaped infundibula (Fig. 1a, b). Worn cusps exhibit bands of brownish dentin lined by enamel ridges (Fig. 1a, b). Sheep molars come into wear while crown elongation still continues cervically (cf. M_2_ in Fig. 1b). The CRB is therefore established late during tooth growth and the crown-root-transition occurs at different times in different locations of an individual tooth, first mesially and distally and last buccally. This is reflected by an undulating course of the CRB with its cuspalmost points located mesially and distally and its apicalmost point buccally (Fig. 1b).

**Fig. 2:**
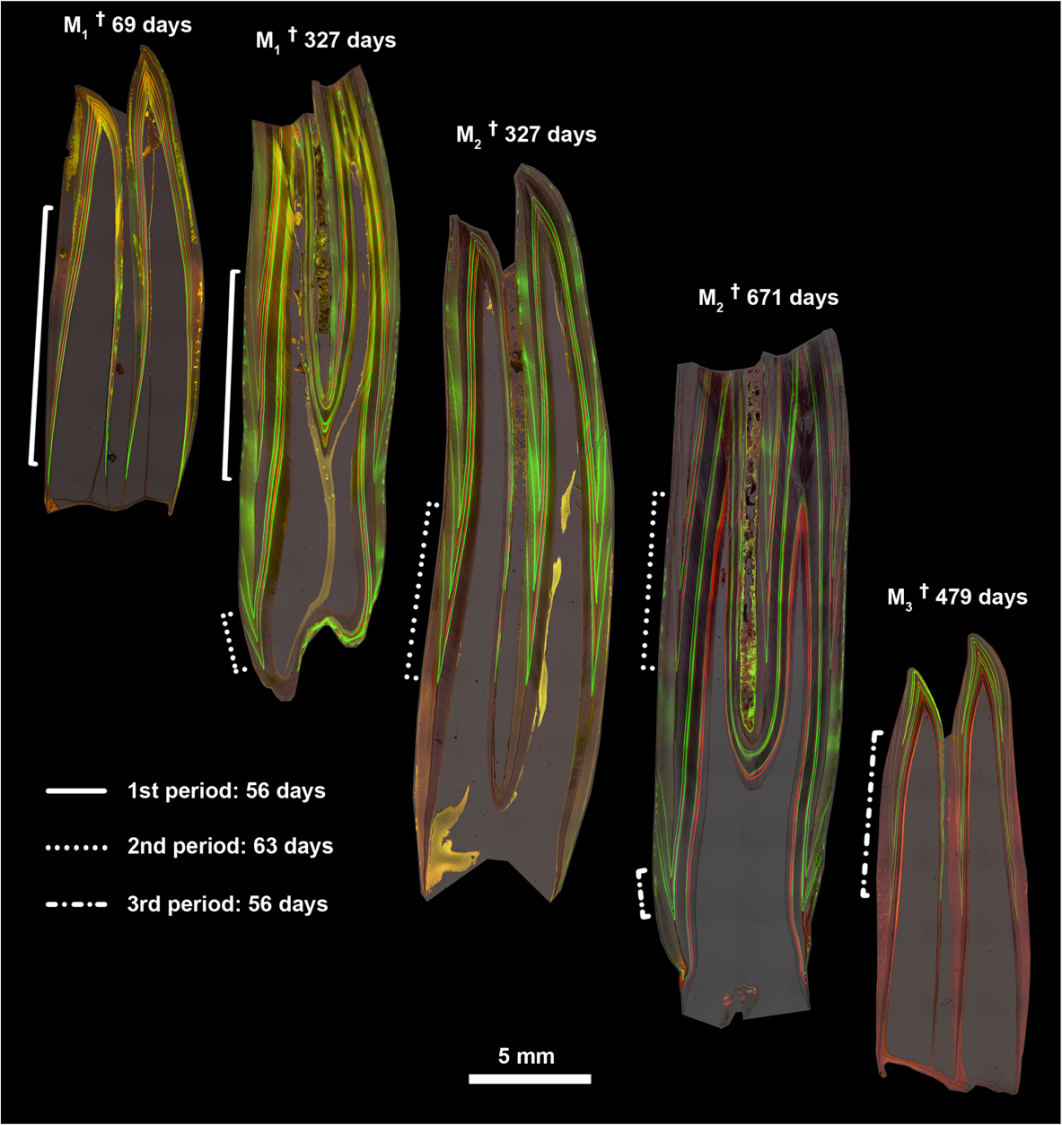
Ground sections of labeled sheep molars. Fluorescent micrographs of ground sections of axiobuccolingually sectioned mandibular molars of Soay sheep repeatedly injected with calcein (green labels) and oxytetracycline (red labels). Crown elongation between consecutive injections during different injection periods (of 56 or 63 days) is indicated by brackets. Note marked differences in extension rate between cuspal and cervical crown portions

Recorded maximum crown height was lower for M_1_ (25.82 mm) than for M_2_ (33.80 mm) and M_3_ (36.03 mm) (Table 2). Crown width of the M_1_ was also lower compared to M_2_ and M_3_ (Table 2). Highest values for the HWR were 4.10 in M_1,_ 4.42 in M_2_, and 4.60 in M_3_ (Table 2). These maxima were calculated for teeth exhibiting initial wear but still ongoing crown formation. A decrease of HWR values occurred after crown elongation had ceased and wear had caused the loss of the cuspalmost crown portions (Table 2, Fig. 2). Regression analysis yielded wear rates of 8.5 μm/day for the M_1_ and of 7.5 μm/day for the M_2_.

**Table 2:**
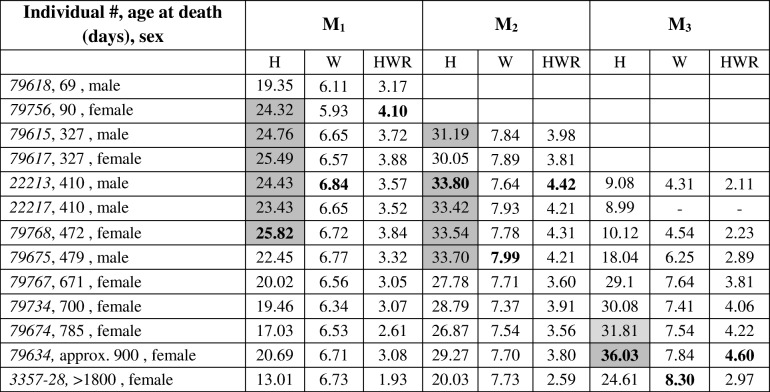
Height (H) and width (W) measurements (mm) of anterior lobes, and HW ratios (HWR) of mandibular molars (M_1_ – M_3_) of Soay sheep at different ages, calculated according to Janis [37]

Bold figures represent maximum values, grey-shaded cells indicate tooth heights ≥90% of the maximum (light grey - 88% in M_3_)

**Fig. 3:**
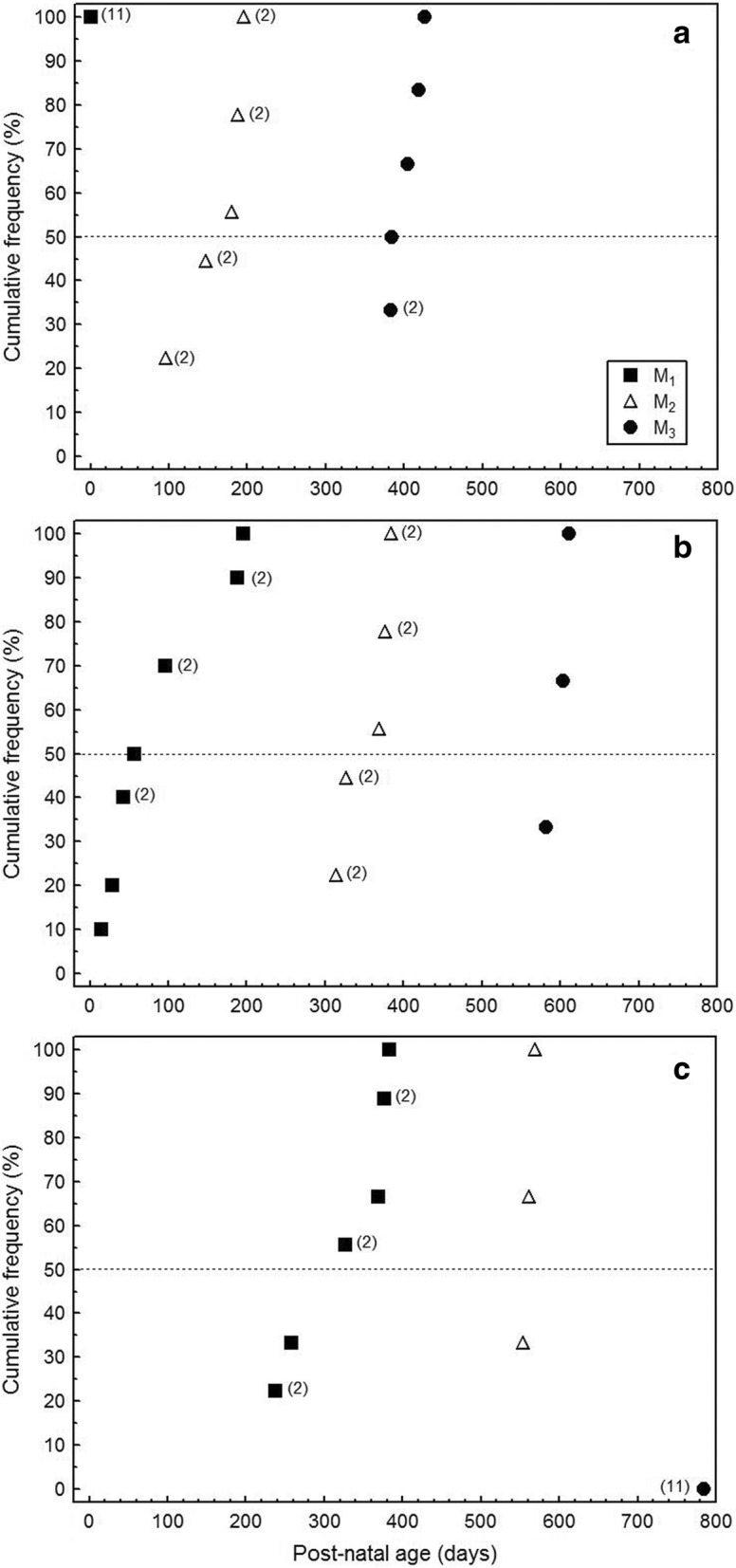
Age at attainment of different developmental stages in mandibular molars. Cumulative frequencies of attainment of different stages during crown development for M_1–3_. (**a**) start of mineralization, (**b**) completion of the infundibular floor, and (**c**) completion of enamel formation on the buccal side of the anterior lobe. Numbers in brackets gives the number of individuals (if > 1) that had achieved the respective developmental stage

Crown mineralization in the M_1_ started already prior to birth. In 50% of the studied M_1_s, formation of the infundibular floor was completed at day 56 of postnatal age, and enamel formation was complete on the buccal side of the anterior lobe at an age of approx. 275 days (Fig. 3a-c). Due to the age composition of our sample and the timing of the fluorochrome injections, completion of the respective stages of crown formation often occurred between two injections. Therefore, the animal ages at which 50% of a molar sample had reached or surpassed a particular developmental stage are considered more informative than the age at which all molars of a sample had reached that stage. In the M_2_, for two developmental stages (start of mineralization and completion of the infundibular floor) these 50% values included teeth that had already considerably progressed beyond these stages. We therefore used the earliest detected label (from injection at day 96, Fig. 3a) to infer the start of mineralization in the M_2_ at about 90 days. In the same way, completion of the infundibular floor in the anterior lobe of the M_2_ was assessed to occur earliest at about 300 days of postnatal age. Enamel formation on the buccal flank of the anterior lobe of the M_2_ was completed at about 560 days (50% value) (Fig. 3a-c). Mineralization of the M_3_ started at an age of about 380 days, and completion of the infundibular floor was achieved at about 590 days (50% values, Fig.3 a,b). Even in the oldest labeled individual (age at death 785 days), M_3_ enamel extension was still ongoing lingually and buccally while it had already been completed mesially (Fig. 3c). The M_3_ of the younger of the two unlabeled individuals included in our study (age at death approx. 900 days) showed the formation of a fused diaphragmatic process at the crown base between the anterior and the central tooth lobe, indicative of a cessation of crown formation in this location. In this specimen, the CRB was already established lingually in the central tooth lobe, while enamel extension was still ongoing buccally. In the posterior lobe of this tooth, enamel extension was still ongoing lingually and buccally.

**Fig. 4:**
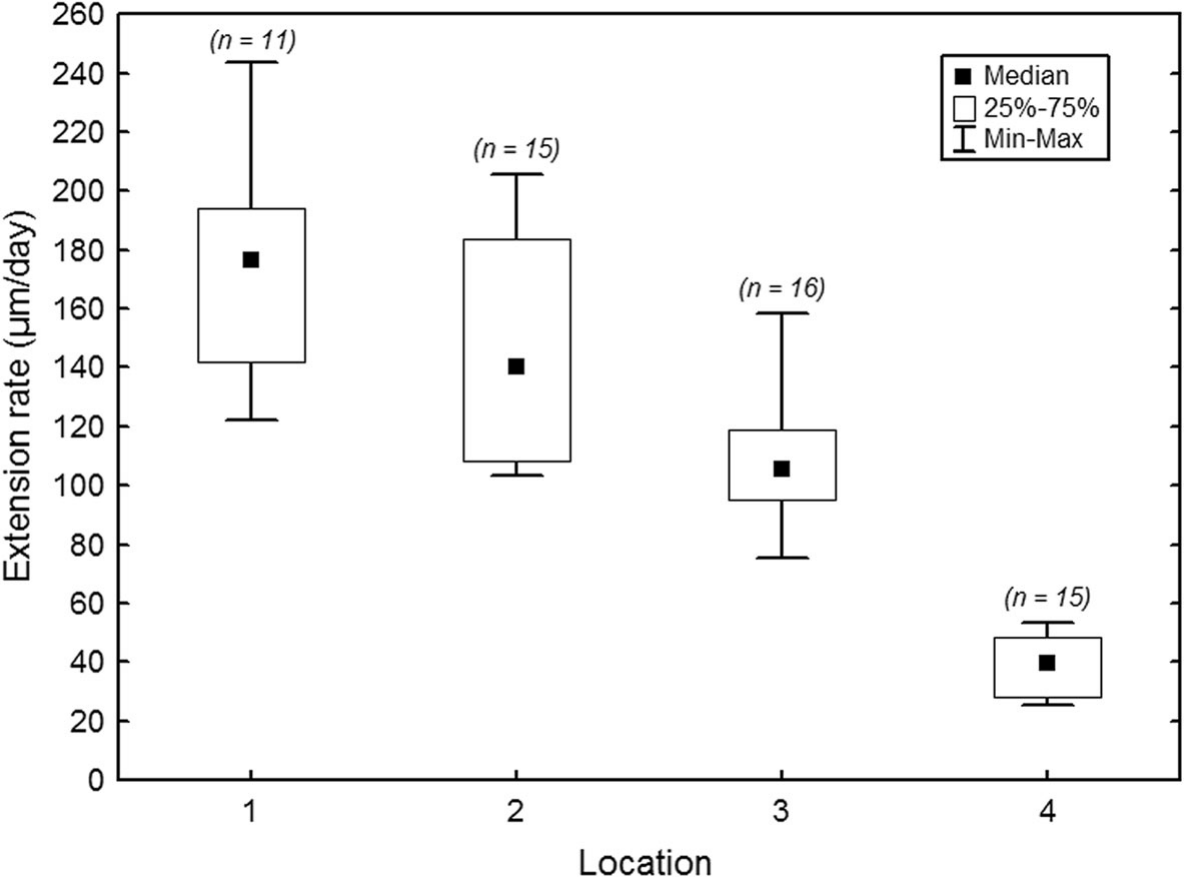
Variation in enamel extension rate of mandibular molars. Enamel extension rates (pooled for the three mandibular molars) recorded in the four quarters of the tooth crown (1: cuspal/upper lateral; 2: mid lateral; 3: lower lateral; 4: cervical). Numbers in brackets: number of measurements in the respective quarters

EERs varied markedly along the vertical tooth axis (Fig. 2). Highest values were recorded in the cuspal/upper lateral quarter of the (reconstructed) total EDJ extension in an unworn complete protoconid (mean EDJ extension: M_1_: 28 mm, M_2_: 33 mm, M_3_: 35 mm; note that for the M_3_ the calculation is based on specimens where crown elongation was still ongoing). Pooled data for all three molars showed a median EER of 176.7 μm/day (range: 122.1–243.3 μm/day) during formation of the cuspal/upper lateral quarter of the EDJ extension. EERs constantly decreased in cervical direction (mid lateral quarter – median: 140.1 μm/day, range: 103.6–205.5 μm/day; lower lateral quarter – median: 105.7 μm/day, range: 75.1–158.4 μm/day; cervical quarter – median: 39.9 μm/day, range: 25.5–53.5 μm/day) (Fig. 4).

**Fig. 5:**
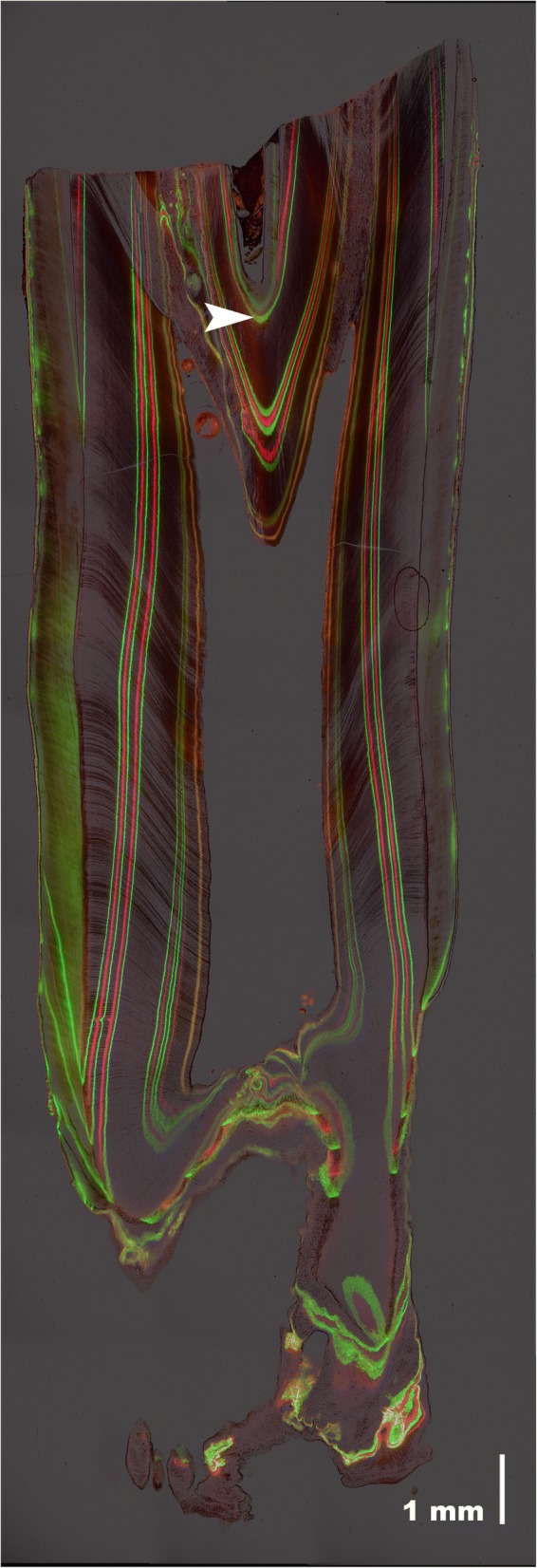
Ground section of a worn mandibular first molar. Fluorescent micrograph of a ground section of the axiobuccolingually sectioned M_1_ of the oldest labeled Soay sheep (individual # 79674). Note large remaining basal crown portion. Arrowhead: floor of the infundibulum. Calcein: green labels, oxytetracycline: red labels

The crown portion located below the level of the infundibular floor (= crown base) accounted for about 40% of the reconstructed total extension of the EDJ of a protoconid in M_1_ (30% in M_2_, not applicable in sectioned M_3_) thus forming a substantial portion of the tooth crown (Fig. 5). After the loss of the infundibulum, presence of enamel is confined to the outer circumference of the tooth crown (Fig. 1a). Due to the asymmetrical extension of enamel at the crown flanks, enamel is eventually only left on the buccal crown flank where the CRB is located furthest apically (Fig.. 5).

## Discussion

Repeated fluorochrome labeling of individuals with forming teeth enables a detailed analysis of tooth growth processes based on a limited number of specimens. Using this approach, we reconstructed molar crown formation in Soay sheep on the basis of 147 developmental stages from a sample of 27 labeled molars of 11 individuals. This allowed us to assess the formative periods of different portions of the molar crowns. While some labels precisely (within a range of a few days) marked the attainment of a characteristic developmental stage, for other stages the labeling pattern did not allow such a precise determination. In these cases, we had to reconstruct the attainment of a particular developmental stage by interpolation. Notwithstanding these limitations, the analysis of fluorochrome-labeled teeth of known-age individuals is a valuable approach for reconstructing dental development. This approach can be viewed as a retrospective longitudinal growth study. Studies with a similar design were previously conducted to characterize crown growth rates and incremental patterns in enamel and dentine of different mammalian specie***s*** [42, 45–50].

A main finding of our study is that molar crown formation in Soay sheep continues for a long time after formation of the infundibular floor and also after the onset of occlusal wear. Molar crown formation covers a period from late fetal development (start of mineralization in the M_1_) to more than 900 days after birth (completion of the crown in the M_3_). Occlusal wear occurred first in molars in which the infundibular floor had been completed and a small amount (3–5 mm) of crown base had already been formed. Therefore, completion of the floor of the infundibulum in mandibular molars can be viewed as a proxy for the onset of tooth eruption (gingival emergence) in sheep.

With the methods used in this study, it was not possible to exactly determine the prenatal onset of enamel formation in the studied M_1_. However, based on mineral apposition rates in dentine we have previously reconstructed the onset of prenatal hard tissue formation in these teeth as occurring between 20 and 40 days prior to birth [50]. This matches quite well with the period of 49 days for prenatal crown formation in the M_1_ recorded for a sheep (Dorset breed) on the basis of lamination counts in prenatal enamel and a calculation of the EER [51]. A study of fetal sheep reported achievement of the bell stage of the M_1_ tooth germ at day 53 of gestation [52]. Hard tissue formation had not yet begun in the oldest fetus (56 days gestational age) of that study. Given a gestation length of approximately 150 days in sheep [53] this suggests a period of at least 50 days between achievement of the bell stage and onset of crown mineralization in the M_1_. Based on our data, between 80 and 100 days elapse between the onset of crown mineralization and the completion of the infundibular floor in the M_1_.

In the anterior lobe of the Soay sheep M_1_, completion of the floor of the infundibulum is followed by a further growth period of approx. 220 days. In the M_2_, in which crown mineralization starts at about three months of postnatal age, about 210 days elapse between the onset of crown mineralization and the completion of the infundibular floor and another 260 days until the crown-root-transition occurs buccally in the anterior lobe. In the anterior lobe of the M_3_, approx. 210 days elapse between the onset of crown mineralization (at 12 to 13 months of age) and completion of the infundibular floor, which is followed by another 300 days of crown elongation prior to the crown-root-transition. In the posterior lobes of M_1_ and M_2_ and the central and posterior lobes of the M_3_, crown mineralization starts slightly later and crown formation lasts slightly longer than in the anterior lobes.

The predominant temporal relationship between crown completion and onset of wear in artiodactyl molars, as schematically illustrated by Hillson [25] for the genera *Cervus* (mesodont condition) and *Bos* (hypsodont condition), is that crown formation is already completed and root formation well under way when the teeth come into wear. The elongated crown base portion of sheep molars is the main difference to the situation in *Cervus* and *Bos*, where the floor of the infundibulum is situated close to the apicalmost point of the CRB (Witzel et al., unpublished observation). The growth pattern of sheep molars differs from that of many other artiodactyl species in that crown elongation continues for a considerable time after the teeth have reached occlusal contact, a condition termed incipient hypselodonty [26]. In addition to sheep ([26, 27] and this study) this pattern also occurs in pronghorns [4, 26, 31], *Myotragus balearicus*, a fossil insular goat species (X. Jordana, personal communication 2015), and in extant and extinct equid species [32, 33].

The extended period of crown growth in hypsodont teeth requires a higher investment compared to the formation of brachydont teeth. The additional investment is partly due to an increased ameloblast number and an extended functional period of the enamel organ. Also, higher numbers of odontoblasts must be recruited and more dentine has to be formed.

An increase in crown height requires the prolonged persistence of an epithelium-derived stem cell niche at the cervical loop of the tooth germ [13]. It has been demonstrated that no novel signaling molecules or pathways have to evolve to alter the shape of a tooth crown or the relative proportions of tooth portions. Rather, changes in tooth size and shape are achieved by varying the temporal expression patterns of genes encoding existing signaling molecules (BMP2 and 4, SHH, IGF, FGF10, Follistatin and Activin) [11, 12]. In ever-growing (hypselodont) teeth, the persistence of the epithelial stem cell niche is likewise linked to the action of different signaling molecules (BMP4, FGF3, 9, and 10, Activin and NOTCH) [11–14]. It may be hypothesized that the same or similar molecules are involved in the prolongation of the crown growth period of sheep molars.

The fallow deer (*Dama dama*) is a cervid species with a body weight (females 35–50 kg, males 50–80 kg [54]) in the range of unimproved breeds of domestic sheep [55]. Body weights of Soay sheep are slightly below that range [56]. Formation time of the mesodont molars [37] of fallow deer is only about half that of the hypsodont molars of Soay sheep (CFT M_1_: *Dama* approx. 150 days – Soay approx. 300 days; CFT M_2_: *Dama* approx. 220 days – Soay approx. 470 days; CFT M_3_: *Dama* approx. 260 days – Soay approx. 500 days) [57, 58]. The data reported for *Dama* tooth development [57, 58] do not allow to differentiate between the formation times of the different crown portions in the M_1_. However, for M_2_ and M_3_ it can be shown that formation time of the crown portion located cuspal to the floor of the infundibulum differs much less between fallow deer and Soay sheep (M_2_: *Dama* approx. 130 days – Soay approx. 200 days; M_3_: *Dama* approx. 180 days – Soay approx. 210 days) than the formation time of the crown base portion (M_2_: *Dama* approx. 90 days – Soay approx. 260 days; M_3_: *Dama* approx. 80 days – Soay approx. 300 days).

The formative periods for the anterior lobes of Soay sheep mandibular molars determined in our study are several months longer than the total crown formation periods reported for modern breeds of sheep on the basis of radiographic and/or macroscopic examinations [27, 59, 60]. These studies also reported an earlier onset of mineralization in the M_2_ (at one month of age) and M_3_ (at 9 to < 12 months) compared to our study in Soay sheep. A reason for this discrepancy could be that in these studies the youngest sheep analyzed were already 3 to 6 [59], 5 [27] or 6 [60] months old so that the onset of M_2_ mineralization had to be extrapolated. In a recent study on Dorset sheep, onset of M_2_ mineralization was first recorded in animals aged 88 days [51]. This value agrees well with our findings for the start of M_2_ mineralization in Soay sheep (at about 90 days). Likewise, the approximate formative periods for the anterior crown lobes of Soay sheep mandibular molars (300 days for M_1_, 460 days for M_2_, and 500 days for M_3_) are only slightly higher than the maximum values given for crown formation of these teeth in Shetland sheep [39].

Differences in reported CFTs between studies can be attributed to two main causes. First, radiographic examination is less well suited for determining the completion of crown formation than histological analysis of labeled teeth or macroscopic inspection of extracted teeth, as has previously been reported for human teeth [61]. Second, molar crown formation in unimproved breeds from Northern Europe takes longer than in more improved sheep breeds that grow at a faster rate [62]. Furthermore, the marked variation in the position of the CRB between different areas of the tooth crown underscores the cautionary remarks by Upex and Dobney [39] about a lack of clarity in many studies with respect to the criteria used to define the developmental stage “crown complete”. Clearly, crown formation is complete only when enamel extension has ceased at all tooth flanks.

The recorded marked reduction of the EER from cuspal to cervical indicates a progressive decline in the recruitment of ameloblasts into the secretory front. Our results corroborate previous findings in sheep [42, 43, 51, 63], other bovids [64, 65] and equids [32–34]. Since tooth wear in sheep occurs simultaneously with the formation of the cervical crown portion, the marked reduction of the EER during later stages of crown formation contributes to a balanced eruption/wear relation, which was described as a characteristic feature of teeth that continue to grow after reaching occlusal contact [28]. However, given the discrepancy between the reconstructed wear rate of about 8.5 μm/day in the M_1_ and the recorded EER of about 40 μm/day in the cervical quarter of the molar crown, further processes must be involved in achieving a balanced relation between eruption and wear. It has been suggested that during the so-called penetrative phase of tooth eruption the teeth become more deeply inserted in the jaw due to a process involving a remodeling of the alveolar process [66].

The estimated M_1_ wear rate of 8.5 μm/day in Soay sheep falls within the range of molar wear rates reported for different grazers (5.6–10.0 μm/day) [67]. Simultaneous crown growth and tooth wear affects the recorded values of the HWR that were highest in sheep that had not yet finished crown formation. Since the HWR of the M_3_ is commonly used as HI [37] in taxon comparisons, it must be considered that HI values for teeth with simultaneous occurrence of crown formation and functional wear are not directly comparable with values for teeth that finish crown formation prior to reaching occlusal contact [68]. The HI of 4.6 recorded by us for Soay sheep is slightly lower than the threshold defining a highly hypsodont condition (> 4.75; sensu Janis [37]) but slightly higher than the values given for *Ovis dalli* (4.08) and *Ovis canadensis* (4.11) [6]. However, assuming that a certain amount of crown reduction (~ 5–10% of total crown height) had already taken place in the M_3_ of the unlabeled Soay sheep with an approximate age of 900 days, a tooth height of about 39 mm can be reconstructed for the unworn M_3_. Dividing this crown height by the measured tooth width of this individual (7.84 mm) results in an HI of 4.97, thus characterizing Soay sheep as highly hypsodont.

The adaptive value of the extended crown formation time in Soay sheep molars lies in the prolongation of their functional period. A first attempt to quantify the relation between relative tooth height and functional period of the molars of different mammalian herbivores was undertaken by Kovalevky in 1874 [9]. He coined the term “Zahnkapital” (tooth capital) to describe the amount of tooth crown available for wear, and related this parameter to the longevity of the respective species.

In a study on the wear stages of mandibular molars of known-age sheep, Moran and O’Connor [69] demonstrated a distinct pattern in the progression of wear recorded with Payne’s reference codes (PRC) [70]. A continuous outer enamel layer as part of the occlusal surface relief is reached at PRC 9A in the first and second molars and at PRC 11G in third molars [70]. In these stages, both lobes of the first and second molars and the anterior and central lobes of the third molar still possess infundibula. After this wear stage is attained (PRC 9A in M_1_ at an age of 12 months and in M_2_ at an age of 26 months, PRC 11G in M_3_ at an age of 32 to 42 months), no further changes in occlusal morphology occur for a considerable period. This is reflected by the fact that individuals of very different ages are included in these wear stages that were therefore termed “persisting stages” by Moran and O’Connor [69]. Of course this “persistence” is only an apparent one, since crown height is progressively reduced with age. The transition to the next wear stage occurs when the infundibulum on the anterior lobe starts to be lost (PRC 12 A in M_1/2_ and PRC 14G in M_3_). After tooth wear has progressed below the level of the infundibular floor, presence of enamel is confined to the circumference of the tooth crown.

Tooth wear patterns similar to those reported by Moran and O’Connor [69] were also recorded for known-age Soay sheep [41]. Ages of Soay sheep exhibiting wear stage 9A in the M_1_ ranged between 12 and 60 months, and between 36 and 108 months in those showing this wear stage in the M_2_. For individuals exhibiting wear stage 11G in the M_3_, an age range of 60–132 months was established. In a study of living, known-age sheep (up to 7 years) from different breeds, Jones [71] reported an age range from 10 to 84 months for the persistence of wear stage 9A in the M_1_. Earliest attainment of this stage in the M_2_ was recorded at 20 months and at 42 months in the M_3_ (wear stage 11G).

Behr [72] studied tooth wear in living, known-age Karakul sheep (age range 3 to 13 years) using a dental replica technique. His detailed description of wear stages allows the assignment of PRCs to the teeth. Loss of the infundibulum in the anterior lobe (PRC 12A) occurred first at 60 months in the M_1_, at 84 months in the M_2_, and at 108 months in the M_3_ (PRC 14G). An earlier loss of the infundibula is reported by Milhaud and Nezit [27] for the anterior lobes of M_1_ (36 months) and M_2_ (72 months) in Pre d’Alp du Sud sheep. According to Behr [72], complete loss of the crown in Karakul sheep, i.e. exposure of root tissue at the occlusal surface was first recorded at an age of 8 years in M_1_ and at 10 years in M_2_. In the oldest specimens of his study (13 years), this stage had not been reached in the M_3_.

Based on the above findings, periods characterized by different occlusal morphologies can be inferred for sheep molars. A maximally functional occlusal morphology with presence of enamel ridges at the buccal and lingual crown flanks and around the infundibulum is present on the anterior lobe of the M_1_ for a period of 24 to 48 months_,_ in the M_2_ for 36 to 54 months, and in the M_3_ for 54 to 60 months. The longer persistence of the infundibulum in the M_3_ compared to M_1_ and M_2_ is probably related to the greater crown height of the former. In addition, a less intense wear of the M_3_ compared to M_1_ and M_2_ may be involved, caused by a higher relative enamel volume in the third compared to the first and second molars that was recorded by Winkler and Kaiser [73] for different mammalian herbivores.

In Soay sheep, the occlusal crown morphology present after the wear plane has progressed below the level of the infundibular floor persists for at least about 4 years in the M_1,_ 5 years in the M_2_ and 6 years in the M_3_. Presence of an extended crown base portion in sheep molars thus results in a markedly prolonged functional period in all molars and, in consequence, an extended maximum lifespan of the individual. In this respect, it is important to realize that enamel formation in the infundibular area is limited to the pre-eruptive period of crown growth because the enamel organ in the infundibular area is lost in the process of tooth eruption. In contrast, enamel formation at the crown base can continue also during the post-eruptive period.

Increasing tooth height and thus the amount of dental tissues, especially enamel, is one of several possible means to increase the functional lifespan of a tooth under an abrasive food regime [4]. Other possible means are an increased enamel hardness [4] and an increased complexity of occlusal enamel ridges [68]. With respect to dental traits, the lack of remodeling in enamel and dentine restricts evolutionary modifications of tooth morphology to the period of odontogenesis [11]. The investment of additional resources into tooth growth, resulting in the prolongation of dental function, thus occurs in advance or partly overlapping with the functional period of the teeth. Within species, dental wear rates have been shown to be correlated with diet composition [74, 75], environmental factors [74], and enamel hardness [76]. These differences also influence longevity, as has been shown for Sika deer (*Cervus nippon*, [74]) and for red deer (*Cervus elaphus*) and wapiti (*Cervus canadensis*) exhibiting dental fluorosis [77, 78]. Increased longevity in populations with greater average molar height has been observed in roe deer (*Capreolus capreolus*) [79].

For *Myotragus balearicus,* comparison of the meso- and macrowear patterns with those of extant mainland artiodactyls suggested a browse-dominated diet and a moderate rate of dental wear [80]. This finding was interpreted as indicating that the abrasiveness of the food was not the only driving force for the increase in crown height, but that the exceptionally long period of tooth formation in this species constitutes a specific adaptation to the conditions of insular life [65, 80, 81]. Resource limitations, low predation pressure, and the adoption of new dietary niches are discussed as being related to a general slowing down of developmental processes in *Myotragus.* There is no evidence for an increased longevity in Soay sheep compared to other (mainland) sheep breeds [82]. Moreover, the crown growth pattern documented here for Soay sheep does not markedly differ from that of mainland sheep [27] or other caprines (domestic goat, mouflon, chamois, Witzel et al., unpublished observation).

## Conclusion

The present study revealed that in sheep molars a long formative period (between 220 days in the M_1_ and 300 days in the M_3_) of the crown bases is related to an extended functional period of these teeth (4 to 6 years) after the wear plane has progressed below the level of the infundibular floor. Our results establish a quantitative link between an additional investment into molar crown growth and the extension of the functional period of these teeth. The reported findings enable an assessment of the adaptive value, in terms of increased longevity, of additional investment into tooth formation.

## Abbreviations

BMP: bone morphogenetic protein
C: calcein
CB: calcein blue
CFT: crown formation time
CRB: crown root border
EDJ: enamel dentin junction
EER: enamel extension rate
FGF: fibroblast growth factor
HI: hypsodonty index
HWR: height width ratio
IGF: insulin-like growth factor
M_1_: mandibular first molar
M_2_: mandibular second molar
M_3_: mandibular third molar
NOTCH: transmembrane receptor protein
PRC: Payne’s reference codes
SHH: sonic hedgehog
T: tetracycline
